# Global quality scores of Brazilian public health system-related YouTube^TM^ videos and their users’ engagement

**DOI:** 10.1590/1807-3107bor-2024.vol38.0099

**Published:** 2024-12-09

**Authors:** Eliane Maria Mascarenhas Silva, Caroline Rabelo Camargos, Isabela Almeida Pordeus, Mauro Henrique Nogueira Guimarães de Abreu, Fabiana Vargas-Ferreira, Flávio Freitas Mattos

**Affiliations:** (a) Universidade Federal de Minas Gerais – UFMG, School of Dentistry, Department of Social and Preventive Dentistry, Belo Horizonte, MG, Brazil.; (b) Universidade Federal de Minas Gerais – UFMG, School of Dentistry, Department of Pediatric Dentistry, Dental School, Belo Horizonte, MG, Brazil.

**Keywords:** Unified Health System, Health Communication, Information Dissemination, Social Media

## Abstract

The study assessed the Global Quality Score (GQS) and informational engagement of users with YouTube videos on the Brazilian public health system (SUS). The YouTube video search tool was used with the Portuguese keywords ‘unified health system’ and ‘SUS’. The first 100 videos returned in the search were studied, using the GQS to measure their educational value, usefulness, and information quality. Users’ engagement with the videos was calculated based on their number of likes/reactions and comments. Other data collected were authorship, year of publication, topic approached, target audience, video length, and use of references. Two trained and calibrated researchers collected the data. Multiple analysis was performed with Logistic Regression, using a 95% confidence interval and significance of p<0.05. There were no poor or generally poor GQS scores (scores 1 and 2) and most videos (58%) achieved moderate or good scores (scores 3 and 4). Videos published after the onset of COVID-19 had a 70% lower chance of engagement than those published in pre-pandemic years (OR: 0.30; 95%CI: 0.12–0.74). Videos that targeted healthcare professionals were 72% less likely to achieve higher GQS scores, than those with an unidentified target audience (OR: 0.28; 95%CI: 0.10–0.75). The informational engagement of the videos showed fewer comments than likes/reactions. Most YouTube videos about the SUS had moderate or good global quality, which was associated with their period of publication and choice of target audience.

## Introduction

The Brazilian Public Health System, named the Unified Health System (SUS) was created as a result of a long struggle for obtaining the universalization and democratization of access to healthcare in Brazil. It recognizes health as a right, allows community participation in public management, and grants people a voice in the construction of their health care.^1^ The media have historically portrayed the SUS negatively, by highlighting issues such as waiting lines, shortage of materials and professionals, and difficulties with accessing health services, and this has perpetuated a harsh public view of the system.^2^

There have been significant changes in the availability of health information, which was previously restricted to health professionals. Nowadays, the Internet is one of the main sources of health information.^3^ Online sources such as Google, Yahoo, YouTube, and Instagram have emerged as prominent channels of public communication.^4^ YouTube is an accessible and popular source of video-delivered health information that eliminates literacy barriers and offers an engaging format. However, as occurs in other social media, unsupervised information posted on YouTube can disseminate low-quality content.^5-7^

Social media users may have difficulty distinguishing between reliable and low-quality information and there has been an increase in the number of studies evaluating the quality and appropriateness of health-related content of YouTube videos.^8^ Assessment instruments have been developed, including the Global Quality Scale (GQS), created to evaluate the educational value, usefulness, and quality of health information on websites.^9^ Subsequently, it has been adapted to evaluate videos^10^ and used in literature to survey the quality of YouTube health-related videos.^11-15^

Considering the quality of health-related information on YouTube as a relevant matter, the aim of this study was to analyze the global quality (GQS) and the engagement with YouTube videos featuring an approach to the Brazilian National Public Health Service (SUS). It also evaluated the dissemination of these videos, by measuring their informational engagement.

## Methods

### Ethical aspects

According to the Brazilian National Health Council Resolution No. 510/2016, there was no need for approval by a Research Ethics Committee, as there was no collection of primary or secondary data on human beings, and only publicly accessible information was collected.

### Study design and data collection

This retrospective cross-sectional study was designed to analyze videos available on YouTube, in Portuguese language, which approached the SUS.

For data collection, a Youtube profile was created, providing mandatory information only. Google Chrome location services were disabled, and its cache was cleared with the aim of reducing data traceability by content display algorithms. The keywords in Portuguese "sistema unico de saude" and "SUS" were used together in the YouTube search (www.youtube.com), and the first 100 videos ranked for relevance by YouTube itself, were collected. The number of users’ reactions/likes and comments on those 100 videos were also collected to measure their engagement^4 16^. Data collection was carried out in May 2022.

The videos included were searched for duplication, lack of audio or images in Portuguese, audio or image errors that made it impossible to watch them, and those that did not address the topic. None of the 100 videos collected from YouTube was excluded from the study. The study variables of interest were collected by two trained and calibrated researchers (EMMS and CRC). Inter-and intra-examiner agreement on data collection were assessed using kappa. The kappa values obtained were 0.545 (intra-examiner 1), 1.0 (intra-examiner 2) and 0.545 (inter-examiner). Agreement was achieved by means of consensus. The videos were characterized by their authorship, year of publication, topic approached, target audience, length in minutes and seconds, and the presence of bibliographic references.

### GQS assessment

**Figure 1:**
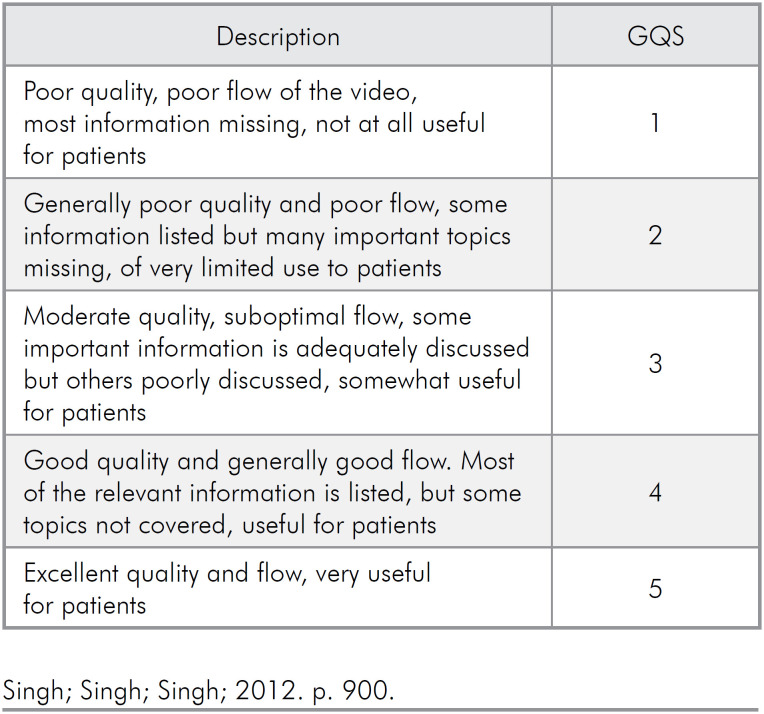
Global Quality Scale (GQS) criteria used to score videos with information about SUS.

To analyze the global quality of the videos, the GQS scale was used (Figure 1). It consists of five sets of criteria and scores that characterize the global quality of videos as poor (score 1), generally poor (score 2), moderate (score 3), good (score 4), or excellent (score 5).^10^

### Assessment of users’ informational engagement

**Figure 2:**
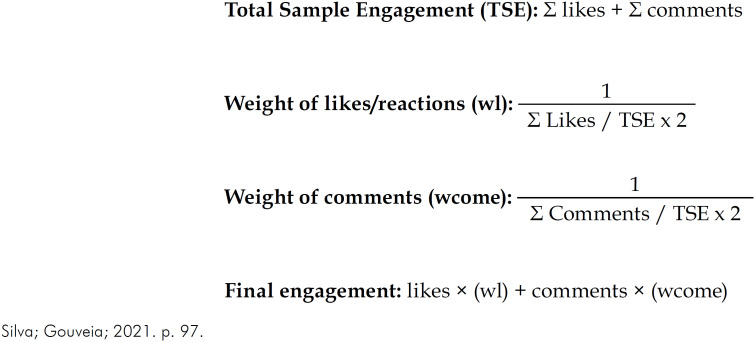
Calculation of users’ engagement with YouTube videos on SUS.

The informational engagement was calculated by using a three-step methodology, developed to analyze likes/reactions, shares, and comments on content published on different social media. YouTube does not allow the identification of shares and allows public access only to comments and likes/reactions. Therefore, to calculate the informational engagement with the videos, only likes/reactions and comments were used^17^ (Figure 2).

The first step of the engagement assessment was to calculate video Total Sample Engagement (TSE), defined by the sum of likes/reactions and comments. The second step was to calculate the weights of both types of users’ interaction with the videos. The contribution of likes/reactions and comments in TSE was weighted to half the sum of each of them divided by TSE. If one of the sums was equal to zero, the weight was considered equal to zero. Finally, the likes/reactions and comments were multiplied by their respective weight, to calculate definition of the final users’ engagement with each video.^17^

### Statistical analysis

Descriptive and multiple analyses were carried out using SPSS version 21.0. The multiple analysis was carried out with unadjusted analysis and adjusted Logistic Regression to evaluate the association between the exposure variables (authorship, year of publication, approached topic, target audience, duration, and use of references) and the outcome variables (informational engagement and global quality). The association measurement was odds ratio (OR) with a 95% confidence interval. Variables with p < 0.05 were considered statistically associated with the outcomes.

After histogram analysis and the use of the Kolmogorov-Smirnov test, the outcomes and the exposure variables showed non-normal distribution. For the multiple analysis, some variables were categorized after watching the 100 videos. They were authorship (teaching, health, and communication institutions/health professionals and students/other professionals and students), topic approached (history and structure/legal norms/challenges and limits), target audience (health professionals and students/unidentified), year of publication (before COVID-19 onset/after COVID-19 onset), and use of references (yes/no). The variable video length was dichotomized by its 50th percentile and the 100 videos were categorized as shorter/longer.

Data on the global quality of YouTube videos was collected according to the five GQS scores: 1 (poor), 2 (generally poor), 3 (moderate), 4 (good), or 5 (excellent)^10^. However, there were no videos with GQS scores 1 and 2 in the study sample. Therefore, for the multiple analysis, the outcome GQS score was categorized as moderate or good (scores 3 and 4) and excellent (score 5). The outcome of the informational engagement was dichotomized by its median into higher and lower.

## Results

Data descriptive analysis is provided in Table 1. Two-thirds of YouTube videos on SUS were authored by teaching, health, and communication institutions or health professionals and students. Although 86% of them did not show the references that the authors used, nearly half (49%) of the videos studied did not have a clear target audience. The only target audience clearly stated in the videos were health professionals and students (51%). The topic most frequently found in the study sample was the history and structure of SUS (53%), which, together with the topic of legal norms, accounted for 80% of the videos. Only 20% of the videos approached the challenges and limits of SUS. There was a large span between shorter (up to 9.49 minutes) and longer videos (between 9.50 and 96.11 minutes) as well as in users’ engagement (from 0 to 36,713). There were only moderate, good, or excellent QGS scores in the study sample.

**Table 1:** Data descriptive analysis.

YOUTUBE		n	%
Year			
	Before COVID-19 (2010 - 2019)	43	43.0
	After COVID-19 (2020 - 2022)	57	57.0
Topic			
	History and structure	53	53.0
	Legal norms	27	27.0
	Challenges and limits	20	20.0
Authorship			
	Other professionals and students	33	33.0
	Teaching, health, and communication institutions	34	34.0
Health professionals and students			
	Unidentified	49	49.0
	Health professionals and students	51	51.0
References			
	No	86	86.0
	Yes	14	14.0
Video length			
	Shorter (up to 9.49 minutes)	51	51.0
	Longer (9.50-96.11 minutes)	49	49.0
Informational engagement			
	Higher (0-491)	52	52.0
	Lower (492-36,713)	48	48.0
GQS			
	Poor	0	0
	Generally poor	0	0
	Moderate	3	3.0
	Good	55	55.0
	Excellent	42	42.0

The video with the highest user engagement addressed SUS legal norms and reached 36,713 likes/reactions (Table 2). SUS challenges and limits was the topic of the 3rd, 4th, 5th, 8th, and 9th videos in the top 10 ranking of informational engagement. Users’ comments were in higher numbers than likes/reactions in the videos in the 4th and 5th positions of the top 10 ranking of informational engagement.

**Table 2:** Top 10 most engaged YouTube videos on SUS and their topics.

Ranking	Total engagement	Likes	Comments	Topics
1	36713	36000	713	Legal norms
2	29032	29000	32	History and structure
3	27465	27000	465	Challenges and limits
4	25898	25000	898	Challenges and limits
5	16634	15000	1634	Challenges and limits
6	15252	15000	252	Legal norms
7	14436	14.000	436	History and structure
8	11114	11000	114	Challenges and limits
9	10301	10000	301	Challenges and limits
10	10242	10000	242	Challenges and limits

In the unadjusted analysis, there was an association between the year of the publication of the videos and users’ engagement (OR: 0.34; 95%CI: 0.15–0.78), which was maintained in the adjusted model. Videos published after the COVID-19 onset had a 70% lower chance of engagement when compared with those published before the COVID-19 onset (OR: 0.30; 95%CI: 0.12–0.74). In the unadjusted analysis, the choice of target audience was associated with the GQS scores of the videos (OR: 0,028; 95%CI 0.12–0.65). After adjusting for potential confounders, videos targeting health professionals and students had a 72% lower chance of achieving higher GQS scores (OR: 0.28; 95%CI 0.10–0.75) when compared with videos with an unidentified target audience (Table 3).

**Table 3:** Crude and adjusted Odds Ratios of the association between users’ engagement to the 100 videos, their Global Quality Scores, and the exposure variables (n = 100), Brazil, 2022.

Variable			Crude model				Adjusted model		
			OR	95%CI	p-value	OR	95%CI		p-value
Engagement								
		Year			0.011				0.009
		Before COVID-19	1.00				1.00		
		After COVID-19	0.34	0.15–0.78		0.30	0.12–0.74		
		Topic						
		History and structure	1.00			1.00			
		Legal norms	0.96	0.38–2.44	0.939	1.17	0.42–3.35		0.763
		Challenges and limits	0.85	0.80–2.39	0.757	0.64	0.21–1.99		0.442
		Authorship						
		Other professionals and students	1.00			1.00			
		Teaching, health, and communication institutions	0.84	0.32–2.21	0.724	0.57	0.19–1.69		0.312
		Health professionals and students	1.63	0.62–4.31	0.326	1.71	0.57–5.12		0.336
		Target audience			0.165				0.108
		Unidentified	1.00			1.00			
		Health professionals and students	0.57	0.26–1.26		0.44	0.17–1.19		
		References			0.872				0.563
		No	1.00			1.00			
		Yes	1.10	0.35–3.40		0.67	0.18–2.55		
		Video length			0.835				0.544
		Shorter	1.00			1.00			
		Longer	0.92	0.42–2.02		1.33	0.53–3		
Global quality score								
		Year			0.230				0.460
		Before COVID-19	1.00			1.00			
		After COVID-19	0.61	0.27–1.36		0.71	0.29–1.74		
		Topic						
		History and structure	1.00			1.00			
		Legal norms	1.05	0.40–2.69	0.923	1.66	0.57–4.80		0.350
		Challenges and limits	1.52	0.54–4.29	0.425	1.24	0.41–3.81		0.702
		Authorship						
		Other professionals and students	1.00			1.00			
		Teaching, health, and communication institutions	1.38	0.52–3.68	0.518	1.24	0.41–3.68		0.704
		Health professionals and students	1.46	0.54–3.91	0.453	1.85	0.61–5.60		0.276
		Target audience			0.003				0.012
		Unidentified	1.00			1.00			
		Health professionals and students	0.28	0.12–0.65		0.28	0.10–0.75		
		References			0.608				0.536
		No	1.00			1.00			
		Yes	0.74	0.23–2.38		0.65	0.17–2.51		
		Video length			0.065				0.424
		Shorter	1.00			1.00			
		Longer	0.47	0.21–1.05		0.69	0.27–1.73		

OR: Odds ratio; CI: Confidence interval.

## Discussion

In recent years, the relevance of social media as health communication tools has become increasingly evident. The high speed at which information is produced and shared raises questions about what is being shared by whom and the quality of information social media users receive. A rapid spread of information of unknown quality, combined with the wide audience, creates an environment prone to the dissemination of misinformation on health-related topics.^18^

In the videos studied, on YouTube SUS was approached mainly by teaching, health, and communication institutions or health professionals and students, with videos frequently targeting health professionals and students. These video authors could target other population groups, especially patients, and use their expertise to deliver good quality SUS-related information. It would be of great use if their videos could help society see beyond the negative portrait of SUS already found in other media.^2^

A previous study on the credibility of YouTube videos emphasized the difficulty with n clearly identifying their target audience and their underuse.^19^ In this study, YouTube videos on the SUS were assessed using the Global Quality Scale (GQS). It reflects their educational value, usefulness, and quality, particularly for patients.^9^ The global quality of the videos studied ranged from moderate to excellent. Similar findings have been reported in other studies that addressed different health issues on YouTube.^11-15^ It was noticeable that YouTube videos about the SUS that targeted health professionals and students had a lower chance of achieving higher GQS scores. It might be that video authors who usually targeted health professionals and students, have not considered that YouTube videos are available to all types of audiences, including SUS patients. Additional care is required if they choose to reach unprofessional audiences, especially patients. For these authors, a step forward would be to use the GQS to evaluate their videos before publication.

Likes or reactions were higher than comments among users’ interactions with SUS-related YouTube videos. The engagement was often used as a metric to evaluate users’ interaction with information on social media, and simple sums to add up likes/reactions and comments might compromise their representation of public engagement.^17^ Likes/reactions express weaker engagement than comments.^20^ Studies on the topic should use weights for the two types of data on engagement to improve understanding of users’ reactions to and actions on the information provided.^21^ The engagement calculation method used in this paper assigned different weights for each type of interaction.

According to literature, political, health, and disaster-related topics raise greater interest among social media users, as they could be associated with their reality. The video with the highest user engagement addressed SUS legal norms. The topic of SUS challenges and limits also reached high users’ engagement. Both item of information should be interpreted in view of the study finding that health professionals and students were the only identifiable target population among the videos in the sample; and those topics were more interesting for their school or job qualification.

The literature has said that, even during the pandemic, Twitter users interacted significantly more with politics and other health-related issues than with the pandemic.^22,23^ Authors have said that controversial issues or topics that generate frequent criticism can lead to higher engagement with social media. The videos on SUS challenges and limits were the most frequent among the Top 10 YouTube videos on SUS that attracted the engagement of users. This type of topic may help video authors to reach and engage with a larger audience that is not restricted to health professionals and students.

Social media fatigue, which describes user exhaustion of accessing social media is caused by the overwhelming amount of information available, and it was on the rise during the COVID-19 pandemic. This phenomenon may have contributed to the observation that videos on SUS posted after the pandemic started attracted less user engagement than those published previously. During global crises user fatigue may be caused by the time spent on social media platforms. In contrast, the use of social media for entertainment tends to lead to addiction, while using it for social purposes tends to improve users’ well-being.^24,25^

This study evaluated the global quality and the informational engagement of users of YouTube videos, associated with variables such as authorship, theme, target audience, length, and use of references. In the same way as occurs with all studies on social media content, it was of a volatile nature and reflected only to the videos available at the time of data collection. To date, this is a pioneering study to address user engagement and quality of information disseminated on YouTube about the Brazilian SUS, national and comprehensive public health system. It offers valuable information for the producers of health system-related YouTube videos and encourages them to design their products with the aim of improving the way people know, understand, and value the Brazilian national public health system.

## Conclusion

The YouTube videos about the SUS analyzed in this study revealed a spectrum of educational value, usefulness, and quality, measured by the Global Quality Scale, ranging from moderate to excellent. Their global quality was negatively associated with the choice of healthcare professionals as a target audience. The informational engagement of users with the videos was negatively affected by the onset of COVID-19, and was predominantly generated by likes or reactions.
